# Disentangling of Malignancy from Benign Pheochromocytomas/Paragangliomas

**DOI:** 10.1371/journal.pone.0168413

**Published:** 2016-12-16

**Authors:** Kyong Young Kim, Jung Hee Kim, A. Ram Hong, Moon-Woo Seong, Kyu Eun Lee, Su-Jin Kim, Sang Wan Kim, Chan Soo Shin, Seong Yeon Kim

**Affiliations:** 1 Department of Internal Medicine, Seoul National University Hospital, Seoul National University College of Medicine, Seoul, South Korea; 2 Department of Internal Medicine, Gyeongsang National University Changwon Hospital, Changwon-si, South Korea; 3 Department of Laboratory Medicine, Seoul National University Hospital, Seoul National University College of Medicine, Seoul, South Korea; 4 Department of Surgery, Seoul National University Hospital, Seoul National University College of Medicine, Seoul, South Korea; 5 Department of Internal Medicine, Seoul National University, Seoul Metropolitan Government Boramae Medical Center, Seoul, South Korea; University of South Alabama Mitchell Cancer Institute, UNITED STATES

## Abstract

**Objective:**

Many malignant tumors initially appear benign but subsequently exhibit extensive metastases. Early identification of malignant pheochromocytomas and paragangliomas (PPGLs) before metastasis is important for improved prognosis. However, there are no robust prognostic indices of recurrence and malignancy. The aim of this study was to identify the clinical and histopathological factors that predict malignant PPGLs.

**Design:**

Retrospective follow-up study.

**Methods:**

In this study, we included 223 patients with pathologically confirmed PPGLs who were treated between 2000 and 2015 at the Seoul National University Hospital in South Korea.

**Results:**

Of these patients, 29 were diagnosed with malignancy, 12 of whom presented with metastatic lesions at the initial diagnosis while 17 developed metastases during follow-up. Nineteen patients with recurrent PPGLs consisted of ones with malignant PPGLs (n = 17) and multifocal PPGLs (n = 2) who had *VHL* and *RET* mutations. The mean age at presentation for malignant PPGLs was significantly younger than that for benign PPGLs (43.0 *vs*. 49.0 years, respectively; *p* = 0.023). Tumor size was not a distinguishing factor between malignant and benign PPGLs (5.0 *vs*. 4.5 cm, respectively; *p* = 0.316) nor did it predict recurrence. Of 119 patients with available pheochromocytoma of adrenal gland scaled score (PASS) data, those with malignant PPGLs presented PASS values ≥4. Of 12 parameters of PASS, necrosis, capsular invasion, vascular invasion, cellular monotony, high mitosis, atypical mitotic figures, and nuclear hyperchromasia were significant predictors of malignancy.

**Conclusions:**

Tumor size did not predict malignancy or recurrence of PPGLs. PPGL patients with characteristic pathologic findings and PASS ≥4 or germline mutations require close follow-up.

## Introduction

Pheochromocytomas (PHEOs) and paragangliomas (PGLs) are rare tumors of the chromaffin tissue that occur in the adrenal medulla or the extra-adrenal glands. PHEOs and PGLs (PPGLs) are not necessarily malignant; however, if metastasis is detected at non-chromaffin sites such as the lymph node or bone, the tumor is reclassified as malignant. Metastasis is the only criterion deeming PPGLs malignant.

Overall, 10–20% of PPGLs are reported to be malignant [1,2]. PPGLs may recur months or years after the initial surgery, and many investigators have suggested predictive factors for recurrent or malignant tumors. There are some reports that younger patients have a higher risk for malignancy [3]. Tumor size has been suggested as a significant risk factor for metastasis, and patients with PGLs rather than PHEOs reportedly tend to have lower survival rates [4]. On the other hand, other studies found that tumor size and primary tumor type have no effect on malignancy rates [5,6]. Malignant PPGLs are identified by the presence of metastases at non-chromaffin sites, and not by local invasion or histopathology [7]. Histologic diagnosis cannot reliably distinguish malignant PPGLs from benign variants. Hence, the ‘pheochromocytoma of adrenal gland scaled score’ (PASS) system was developed, although it has been criticized for its limitations [8,9]. Norepinephrine or dopamine secretion was also reported to be predictive of malignancy; however, its discriminatory power is weak [10]. Recently, germline mutations in the *SDHB* and *SDHD* genes have been reported to be independent risk factors for metastasis [11]. Moreover, the *SDHB* gene is mainly associated with PGLs and tends to predict increased aggressiveness and lower survival rates [12,13]. However, the known predictive factors for malignancy are not reliable; therefore, it is recommended that all patients receive life-long follow-up after the initial surgery [14].

In the present study, we aimed to identify the clinical, histopathological, and genetic factors that predict benign vs. malignant PPGLs; this would be particularly helpful for predicting whether PPGLs that are initially diagnosed as benign will subsequently become malignant.

## Subjects and Methods

The present study included 223 patients with pathologically confirmed PPGLs (PHEO, n = 145; PGL, n = 78) post-surgery, who were treated between 2000 and 2015 at the Seoul National University Hospital in South Korea. All patient data were collected from medical records. Clinical information included i) sex and age at initial diagnosis; ii) clinical symptoms/signs including headache, sweating, palpitation, pain (neck, chest, abdomen, or bone), palpable mass, and hypertension (new onset, paroxysmal, or uncontrolled); iii) secreted hormones (plasma/24 hours urine): epinephrine, norepinephrine, metanephrine, normetanephrine and dopamine; iv) tumor size; v) PASS; vi) genetic mutations; vii) recurrence; and viii) metastasis.

Catecholamine type was classified according to Kimura et al. [15]. Elevated epinephrine or metanephrine was defined as the epinephrine type regardless of norepinephrine levels. Elevated norepinephrine or normetanephrine, irrespective of high levels of dopamine, was defined as norepinephrine. The PASS system was proposed by Thompson to distinguish between benign and malignant PHEO [5]. PASS is based on assessing 12 histologic parameters: necrosis, capsular invasion, vascular invasion, extension into the adipose tissue, large nests or diffuse growth, high cellularity, cell spindling, cellular monotony, mitotic figures (>3/10 high-power field [HPF]), atypical mitotic figures, nuclear pleomorphism, and nuclear hyperchromasia. Tumors with a PASS >4 reportedly exhibit more malignant tendencies [5,16]. Tumor size and PASS data were obtained from pathologic records.

Preoperative imaging tests were performed to identify and locate PPGLs. In accordance with the JCEM guideline, we used computed tomography (CT) rather than magnetic resonance imaging (MRI) as the first-choice imaging modality [14]. MRI was recommended in patients with detection of skull base and neck PPGLs, surgical clips that cause artifacts, an allergy to CT contrast, and in patients who need limited radiation exposures [14]. In the present study, CT was performed in all patients, and located PPGLs. ^123^I-metaiodobenzylguanidine (MIBG) scintigraphy was used as a functioning imaging modality to detect metastasis in patients with large size of the primary tumor or to extra-adrenal, multifocal or recurrent PPGLs.

Recurrence was defined as the reappearance of disease after elimination of the tumor, as confirmed by biochemical and imaging tests [17].

According to the World Health Organization classification, malignant PPGL is defined as the presence of metastases in non-chromaffin organs that are distant from the primary tumor such as the lung, mediastinum, kidney, bone, liver and spleen [7]. If such metastatic lesions are discovered in a patient during follow-up, the primary tumor is categorized as malignant, irrespective of the initial report.

Mutation screening, including for all exons of *VHL*, *SDHB*, and *SDHD* as well as exons 8, 10, 11, and 13–16 of the RET gene, was performed by direct sequencing. Multiplex ligation-dependent probe amplification was conducted for *VHL*, *SDHB*, and *SDHD* [18].

The present study was approved by the institutional review board of Seoul National University Hospital (IRB No. 1606-104-771) and performed in accordance with the Declaration of Helsinki. The informed consents from study subjects were waivered due to the retrospective study design.

### Statistical analysis

Data are shown as mean ± standard deviation for parametric continuous variables, median [range] for nonparametric continuous variables, or n (%) for categorical variables. Student’s *t* test (parametric data) and the Mann-Whitney U-test (nonparametric data) were used for continuous variables to compare the clinical and pathological characteristics of patients with PHEO *vs*. PGL, or of those with benign *vs*. malignant PPGLs. Categorical variables were analyzed by using the χ^2^ test. Logistic regression models were performed to identify clinical and pathological parameters predicting recurrent and malignant PPGLs. Statistical analyses were performed using SPSS version 18 (IBM, SPSS Inc., Chicago, IL, USA). *P* < 0.05 was considered statistically significant.

## Results

Clinical characteristics of patients with PHEOs and PGLs are shown in **Table 1**.

**Table 1:** Clinical and pathological characteristics of patients with PHEO and PGL.

	All (n = 223)	PHEO (n = 145)	PGL (n = 78)	*p* value
Age at diagnosis (years)	48.0 [37.0–59.0]	46.0 [35.5–58.0]	50.5 [37.0–60.3]	0.270
Male/Female	112/111	71/74	41/37	0.608
Type of presentation				
Headache (%)	46 (20.6)	38 (26.2)	8 (10.3)	**0.005**
Sweating (%)	39 (17.5)	33 (22.8)	6 (7.7)	**0.005**
Palpitation (%)	50 (22.4)	45 (31.0)	5 (6.4)	**<0.001**
Neck, chest, abdomen, bone pain (%)	33 (14.8)	20 (13.8)	13 (16.7)	0.564
Palpable mass (%)	9 (4.0)	1 (0.7)	8 (10.3)	**0.001**
Hypertension (%)	72 (32.3)	57 (39.3)	15 (19.2)	**0.002**
Hypertension (%)	65 (29.2)	45 (31.0)	20 (25.6)	0.398
Diabetes (%)	38 (17.0)	28 (19.3)	10 (12.8)	0.219
Catecholamine type	N = 173	N = 139	N = 34	**<0.001**
Epinephrine (E or E+NE)	82 (47.4)	73 (52.5)	9 (26.4)	
Norepinephrine (NE or NE+DA)	63 (36.4)	52 (37.4)	11 (32.4)	
Nonfunctioning	28 (16.2)	14 (10.1)	14 (41.2)	
Tumor size (cm)	4.8±2.8	5.1±2.9	4.4±2.5	0.067
PASS (point)^a^	3.0 [2.0–5.0]	3.0 [1.0–5.0]	3.0 [2.0–6.5]	0.578
PASS > 4 points (%)	52 (43.7)	38 (42.2)	14 (48.3)	0.568
Germline mutation (%)^b^	33 (33.0)	29 (31.9)	4^c^(44.4)	0.444
Metastasis at diagnosis (%)	12 (5.3)	4 (2.8)	8 (10.2)	**0.018**
Malignancy (%)	29 (13.0)	13 (9.0)	16 (20.5)	**0.014**
Recurrence (%)^c^	19 (9.0)	11 (7.8)	8 (11.4)	0.446

Data are shown as median [range], mean ± standard deviation, or n (%).

^a^ available in PHEO (n = 90) and PGL (n = 29)

^b^ available in PHEO (n = 91) and PGL (n = 9), included one patient with both PHEO and PGL

^c^ included only PPGLs in remission (PHEO, n = 141; PGL, n = 70)

Of the 223 patients, 145 were diagnosed with PHEOs and 78 with PGLs. One patient had concurrent PHEO and PGL. There was no bias towards either PHEO or PGL according to patients’ age or sex. PHEO patients exhibited more symptoms than those with PGL. The mean PPGL tumor size was 4.8 ± 2.8 cm. PASS data were available for 119 patients (PHEO, n = 90; PGL, n = 29); the median score was 3.0 points (range, 2.0–5.0 points). Tumor size and PASS were similar for PHEOs and PGLs. Among 100 patients who underwent germline mutation analysis, there was no significant difference in mutation rate between PHEO and PGL patients. The median follow-up duration for the patients after surgery was 38.0 months (range, 18.5–79.2 months). Of 223 patients, 29 (13%) were diagnosed with malignancy either at diagnosis or during follow-up, and PGLs behaved more aggressively than PHEOs (PHEO, n = 13 [9.0%]; PGL, n = 16 [20.5%]; *p* = 0.014). Twelve of 29 patients presented with metastatic lesions at the initial diagnosis, whereas 17 patients (PHEO, n = 9; PGL, n = 8) developed metastasis at recurrence. **Table 2** shows clinical and pathologic characteristics of patients with benign and malignant PPGLs.

**Table 2:** Clinical and pathologic characteristics in patients with benign and malignant PPGLs.

	Benign (n = 194)	Malignancy (n = 29)	*p* value
Age at diagnosis (years)	49 [38–60]	43 [30–52]	**0.023**
Female (%)	97 (50.0)	14 (48.3)	0.862
Tumor type			**0.014**
PHEO	132 (68.0)	13 (44.8)	
PGL	62 (32.0)	16 (55.2)	
Catecholamine type	N = 160	N = 13	0.368
Epinephrine (E or E+NE)	78 (48.8)	4 (30.8)	
Norepinephrine (NE or NE+DA)	56 (35.0)	7 (53.8)	
Nonfunctioning	26 (16.2)	2 (15.4)	
Tumor size (cm)	4.5 [2.9–6.3]	5.0 [3.3–7.5]	0.316
≥ 5 cm	81 (41.8)	15 (51.7)	0.323
PASS (points)	N = 109	N = 10	
median	3 [1–5]	8 [6–10]	**<0.001**
≥4 points, case (%)	42 (38.5)	10 (100)	**<0.001**
necrosis (%)	9 (8.3)	7 (57.1)	**<0.001**
capsular invasion (%)	44 (40.4)	9 (90)	**0.005**
vascular invasion (%)	17 (15.6)	8 (80)	**<0.001**
extension into the adipose tissue (%)	21 (19.3)	3 (30)	0.420
large nests or diffuse growth (%)	24 (22.0)	3 (30)	0.693
high cellularity (%)	41 (37.6)	3 (30)	0.743
cell spindling (%)	5 (4.6)	1 (10)	0.416
celluar monotony (%)	8 (7.3)	3 (30)	0.050
mitotic figures (>3/10 HPF) (%)	7 (6.4)	3 (30)	**0.0380**
atypical mitotic figures (%)	3 (2.8)	3 (30)	**0.008**
nuclear pleomorphism (%)	40 (36.7)	6 (60)	0.182
nuclear hyperchromasia (%)	23 (21.1)	6 (60)	**0.013**
*SDHD* mutation	4/91	0/9	0.521
*SDHB* mutation	2/91	0/9	0.653
Recurrence	2 (1)	17 (58.6)	**<0.001**

Data are shown as median [range] or n (%).

**Fig 1:**
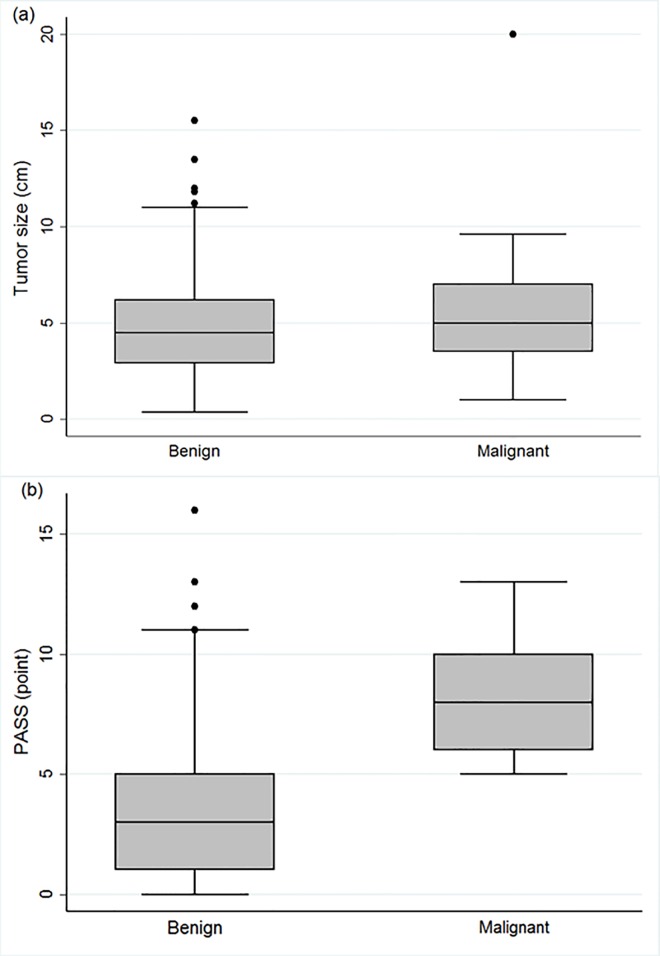
Distribution of (a) tumor size and (b) PASS in patients with benign and malignant PPGLs.

The mean patient age at presentation for malignant PPGLs was significantly younger than that for benign PPGL (43.0 *vs*. 49.0 years respectively; *p* = 0.023). However, no preponderance according to sex was observed. Tumor size was not a distinguishing factor between malignant and benign PPGLs (5.0 [3.3–7.5] *vs*. 4.5 [2.9–6.3] respectively; *p* = 0.316) (**Fig 1A**). The proportion of patients with a tumor size over 5 cm was similar in those with benign and malignant lesions. The same results were reported in subgroup analyses according to PHEOs and PGLs (data not shown). PASS data were only available for 119 patients (benign, n = 109; malignant, n = 10). Median PASS values for malignant PPGLs were significantly higher than those for benign tumors (8 [6–10] *vs*. 3 [1–5] points respectively; *p*<0.001). All patients with malignant PPGLs showed PASS ≥4, while 42 (38.5%) of benign PPGL patients also presented with PASS ≥4. All PPGLs with PASS values <4 had benign clinical courses without recurrence or malignancy regardless of tumor size (**Fig 1B**). Of the investigated 12 parameters of PASS, necrosis, capsular invasion, vascular invasion, cellular monotony, high mitosis (>3/10 HPF), atypical mitotic figures, and nuclear hyperchromasia were significantly associated with malignancy.

We compared clinical and pathological variables in benign (n = 194) *vs*. malignant PPGLs (n = 17) in initially “benign” PPGLs (n = 211) (**Table 3**).

**Table 3:** Logistic regression models predicting malignancy in patients with initially “benign” PPGLs.

Variables	Odds Ratio (95% CI)	*P*-value
Age ≤ 35	4.2 (1.5–11.6)	**0.005**
PGLs	4.7 (0.9–22.5)	0.052
Tumor size ≥ 5cm	0.6 (0.1–2.6)	0.445
PASS (1point)^a^	1.4 (1.1–1.7)	**0.001**
Necrosis	9.0 (2.8–28.3)	**<0.001**
Capsular invasion	5.3 (1.4–19.7)	**0.013**
Vascular invasion	11.2 (3.2–38.9)	**<0.001**
Cellular monotony	9.5 (1.8–50.0)	**0.008**
Mitotic > 3/10 HPF	11.1 (2.9–42.8)	**<0.001**
Atypical mitotic figures	21.2 (4.1–110.5)	**<0.001**
Nuclear hyperchromasia	6.0 (1.3–26.8)	**0.020**

CI, confidence interval

^a^, available in 109 benign and 7 malignant and recurrent PPGLs due to lack of PASS.

Logistic regression models to determine the parameters that are predictive of malignancy were performed including only initially benign PPGLs. According to these models, being <35 years of age was significantly associated with a 4-fold higher risk of malignancy. PASS data were available for 116 patients with initially benign PPGLs; in these patients, necrosis, capsular invasion, vascular invasion, high mitosis, atypical mitotic figures, and nuclear hyperchromasia were predictive factors for malignancy.

Nineteen of 211 cured patients developed recurrence during a median follow-up duration of 38 months; the recurrence rate was 9.0%. Two of these patients, both of whom had *VHL* and *RET* mutations, experienced recurrence at the adrenal gland; this was not a criterion for malignancy. Of the 17 remaining patients, 1 had only regional intra-abdominal lymph node metastasis. Five patients had single organ metastasis to the spleen, lung, mediastinum, bladder, and bone, respectively; the remainder had multiple distant metastases. The median time to recurrence was 38.7 months (range, 10.0–74.3 months) (**Table 4**).

**Table 4:** Characteristics of PPGL patients with recurrence.

No	Gender	Diagnosis	Age (years)	Size (cm)	PASS	Cateholamine-producing	Mutation	Recur site	Time to recurrence (month)
1	Female	PHEO	11	4	N/A	(+)	VHL	locoregional	44
2	female	PHEO	24	2	N/A	(+)	RET	locoregional	38.6
3	Male	PHEO	13	3.8	N/A	(+)	N/A	Bone, LN (portocaval)	15.7
4	Male	PHEO	10	3.5	N/A	(+)	VHL	Spleen	103.5
5	Male	PHEO	23	4.8	10	(+)	RET	Spleen, locoregional	46.7
6	Male	PHEO	54	7.0	13	(+)	N/A	Scalp, Lt.kidney	6.9
7	Male	PHEO	52	5.0	N/A	(+)	(-)	Multiple abdominal LN, locoregional	80.8
8	Female	PHEO	10	1.2	8	(+)	VHL	Multiple abdominal LN, locoregional	74.3
9	Female	PHEO	33	3.0	N/A	(+)	RET	LN (portocaval)	17.3
10	Female	PHEO	46	8.5	N/A	(+)	(-)	Mediastinum, locoregional	61.1
11	Female	PHEO	31	3.0	N/A	(+)	N/A	LN(aortocaval), locoregional	22.3
12	Male	PGL	34	7.9	N/A	(+)	N/A	Lung	99.9
13	Male	PGL	58	8.0	10	(+)	N/A	Bone, multiple abdominal LN	2.3
14	Male	PGL	54	5.5	N/A	(+)	N/A	Liver, lung	77.8
15	Female	PGL	40	1.6	N/A	(+)	N/A	Mediastinum	30.8
16	Female	PGL	18	1.0	N/A	(+)	N/A	Bladder	10.0
17	Female	PGL	29	5.5	6	(+)	(-)	Bone	4.7
18	Female	PGL	35	4.5	6	(+)	N/A	Liver, multiple abdominal LN	68.8
19	Male	PGL	53	5.6	7	(-)	(-)	Bone, LN(parapharyngeal), sacrum	6.0
			33 [18–52]	4.8 [3.0–7.0]					38.7 [10.0–74.3]

N/A, not applicable

Two patients with malignant PPGLs expired 4.13 and 5.15 years after initial diagnosis, respectively, during a follow-up duration of 6.48 years.

## Discussion

The present study showed that the malignancy rate was 13.0% (29/223), while the recurrence rate was 9.0% (19/211). Malignant PPGLs presented at a younger age than benign tumors. Primary tumor size did not significantly differ between benign and malignant PPGLs. The PASS was lower in benign than in malignant PPGLs; all malignant PPGLs had a PASS of ≥4 points. *SDHB* mutation did not predict malignancy, whereas 12 variables of PASS, necrosis, capsular invasion, vascular invasion, cellular monotony, high mitosis, atypical mitotic figures, and nuclear hyperchromasia were predictive of malignancy in PPGLs that did not present with metastases at diagnosis.

Previous studies have reported that the malignancy rate was approximately 10% for PHEOs and 15–35% for PGLs [19]. Similarly, 9.0% of patients with PHEOs and 20.5% of those with PGLs in our study developed metastatic lesions during the median 38.0 months of follow-up. Another study revealed that recurrence of abdominal PGL can occur in nearly 25% of patients after complete resection [20]. In our study, PPGL recurrence occurred in 19 patients (8.5%; PHEO, n = 11; PGL, n = 8). Two patients had recurrence in their adrenal glands, which was not a criterion for malignancy; these patients also had germline mutations. In all cases, recurrent tumors developed within 10 years. However, it has been reported that recurrence or metastasis can develop several decades after surgery [21]. As recommended by *European Society of Endocrinology* guideline, only high-risk patients such as those who are young and those with germline mutations and large tumors should be followed for more than 10 years. Skull base and neck paragangliomas can be often biochemically negative and only imaging tests can detect tumors. Thus the guideline suggest performing imaging tests every 1-2years in nonfunctioning PPGLs and assaying plasma or urinary catecholamines or metanephrines annual follow-up in functioning PPGLs [14,22].

A younger age at diagnosis has been suggested to be a clinical risk factor for malignant PPGLs. Although Feng et al. reported no age difference between patients with benign *vs*. malignant PPGLs, [23] our present study showed that malignant PPGLs occurred more often in patients of younger age than in older patients, although the high-risk age cut-off had not been determined. The mean age of disease onset in our study was relatively old, considering that the peak age of PPGL incidence is in the fourth decade [3]. According to our results, patients aged less than 35 years should be followed carefully due to their 4.2-fold higher risk for malignancy, even those with no metastasis present at diagnosis.

We demonstrated that malignant tumors were more likely to be PGL than PHEO (55.2% *vs*. 44.8% respectively; *p* = 0.014). PGLs, particularly sympathetic, intra-abdominal lesions, exhibit a higher rate of malignancy and recurrence than do PHEOs according to previous studies [3,4]. However, PGLs that initially appeared to be benign did not tend to recur as malignancies in the present study.

Some studies have suggested that primary tumor size may indicate malignant behavior in PPGLs [4,24–26]. O’Riordian et al. suggested that a tumor size >5 cm was a strong predictor of malignancy, although they included only PGLs in their study. Meanwhile, Agarwal et al. advocated a cut-off size of 6 cm for discriminating tumors at a high risk of malignancy [26,27]. Ayala-Ramirez et al. acknowledged a wide variability in size between malignant and benign PPGLs, and advised follow-up even for patients with small tumors [4]. We were unable to determine a cut-off size for predicting malignancy because of the overlap in size between benign and malignant PPGLs.

The PASS was proposed as a scoring system for malignancy risk in 2002 [5]. However, there have been conflicting reports regarding its reliability in predicting malignancy in PHEOs [16,27]. Thompson et al. found that tumors with a PASS >4 exhibited increased metastatic potential [5,16]. In the present study, there were no malignant PPGLs in patients with PASS <4 points, which may indicate that PPGLs in patients with a PASS <4 tend to be benign. However, the PASS has been criticized for its poor concordance with several parameters used by expert pathologists; moreover, the system was not developed for PGLs [9]. A more recent scoring system, the Grading of Adrenal Pheochromocytoma and Paraganglioma (GAPP), was developed by Kimura et al. in 2014. Several PASS parameters including large nests, cellularity, necrosis, and capsular and vascular invasion were incorporated into the GAPP. Consistent with this, we found that necrosis and capsular and vascular invasion were significant predictors of malignant recurrence in patients with initially “benign” PPGLs, as well as those who presented with metastases at diagnosis. Furthermore, high mitotic figures, atypical mitotic figures, and nuclear hyperchromasia were additional predictors of malignancy, as shown in other studies [15,16]. Interestingly, we did not find any correlation between the PASS and tumor size. As such, a high PASS must be considered to be strongly associated with malignant potential, but is not diagnostic of malignancy.

Norepinephrine- or dopamine-producing tumors have a higher risk of malignancy; these parameters were incorporated into the GAPP system [10,15]. However, our study did not show that norepinephrine-producing tumors tended to be more malignant compared to epinephrine-producing tumors. Nineteen patients with recurrent PPGLs included those with malignant PPGLs (n = 17) and multifocal PPGLs (n = 2) who had *VHL* and *RET* mutations. The presence of an *SDHB* mutation is associated with an increased risk of metastasis, and up to 40% of patients with an *SDHB* mutation will develop distant metastasis [13]. Because of the lack of genetic testing in malignant PPGLs, we did not investigate the proportion of *SDHB* mutations in malignant PPGLs.

Our study had several limitations. First, it was a retrospective analysis based on medical records; hence, several data may inevitably have been unavailable. Only a portion of the patients had PASS and gene mutation data available. Second, we did not assess Ki-67 indexes, and GAPP scores could not be calculated in the present study. Third, some of benign PPGLs can be classified into malignancy if the patients are followed-up longer. The follow-up duration was relatively shorter in patients with non-metastatic PPGLs (n = 17) than those with metastatic ones (n = 194) (34.0 [18.1–69.0] vs. 85.3 [49.6–100.2], *p* value = 0.009).

However, our study includes certain strengths. Despite the rarity of PPGLs, we collected a relatively large sample size at a single center, and we comprehensively reviewed these patients’ clinical, pathological, and genetic data. Notably, we focused on metachronous malignant PPGLs that initially presented as benign but later recurred as metastatic lesions, and analyzed and validated each of the parameters of the PASS in addition to the total scores.

In conclusion, tumor size does not predict PPGL malignancy or recurrence; however, PPGL patients younger than 35 years of age and/or those with PASS ≥4 points require close follow-up. PPGLs with PASS <4 have a benign clinical course without recurrence or malignancy regardless of tumor size. Further investigation is required to elucidate novel and powerful markers for malignant PPGLs.
